# Ageing medical workforce in Australia - where will the medical educators come from?

**DOI:** 10.1186/1478-4491-7-82

**Published:** 2009-11-05

**Authors:** Deborah J Schofield, Susan L Fletcher, Emily J Callander

**Affiliations:** 1 Northern Rivers University Department of Rural Health, School of Public Health, Faculty of Medicine, University of Sydney, Syndey, Australia

## Abstract

**Background:**

As the general practitioner and specialist medical workforce ages there is likely to be a large number of retirees in the near future. However, few Australian studies have specifically examined medical practitioner retirement and projected retirement patterns, and the subsequent impact this may have on training future health care professionals.

**Methods:**

Extracts from the Australian Medicare database and Medical Labour Force Surveys are used to examine trends in attrition of general medical practitioners and specialists over the age of 45 years from the workforce and to predict their rate of retirement to 2025.

**Results:**

The general medical practitioner workforce has aged significantly (p < 0.05). Between the years 2000 and 2025, it was projected that 43% of the year 2000 general practitioner workforce and 56% of the specialist workforce would have retired.

**Conclusion:**

The ageing of the baby boomer and older cohorts of the general practitioner and specialist workforce will lead to a significant number of retirements over the next 20 years. Increasing the numbers of students and new medical schools has been heralded as a means of alleviating service shortages from about 2015 onwards; however, the retirement of a large proportion of experienced health care professionals may lead to shortages of educators for these students.

## Background

Two major government reports have examined the impact of ageing in Australia - the Intergenerational Reports (IGR) released by the Treasury in 2002 and 2007 [1,2] and the report on the Economic Implications of an Ageing Australia by the Productivity Commission [3]. These two reports highlighted future pressures which threaten the sustainability of the Australian Government budget balance due to the growing needs of an ageing population and labour shortages which will limit economic growth and taxation revenue. In addition, ageing of the 'baby boomer' generation was found to increase demand for health care, and consequently the ageing population will require more health professionals to deliver the services required in an environment where the existing workforce is moving rapidly towards retirement.

Numerous studies have noted ageing of the Australian medical workforce[4-7]. However, there has been very little research specifically on general medical practitioner or specilaist retirement or retirement intentions in Australia. One recent study which examined ageing and rates of retirement amongst the general practitioner and registered nursing workforce [8] concluded that there will be a period of rapid retirement from the medical workforce over the next 15 years. A recent paper projecting Australian medical workforce supply from 2001-2012 [9] included the impact of ageing and concluded that there will not be enough doctors in 2012. This has contributed to calls for more medical training places [10] to both meet the increasing demand for health care caused by the ageing Australian population, and to overcome the loss of medical practitioners as the medical workforce ages. The government has responded by significantly expanding the number of medical places available.

This paper will examine ageing of the general medical practitioner and specialist workforce in Australia and will project the numbers and timing of their retirement to 2025. The impact that the retirement of experienced health care professionals has on the training requirements of the future health care workforce will be discussed.

## Methods

The methods used in this paper are similar to those used recently in Australia to examine past general practitioner and nursing retirement [8] but using different data sources and projecting the patterns of retirement into the future.

Grouped data on demographic characteristics (age and sex) were obtained for general practitioners from the Australian Government Department of Health and Ageing's Medicare data and for specialists (age, sex and hours worked), from the Australian Institute of Health and Welfare's (AIHW) annual Medical Labour Force Surveys. The Medicare data was provided from 1984-85 to 2004-05 and the Medical Labour Force Survey data from 1995 to 2003. The census was conducted before 1995, but the definitions for specialists have changed substantially so that comparisons with earlier years are not reliable.

Using Medicare and Medical labour force survey data, 5-yearly cohorts of general and specialist medical practitioners aged 45 years and over were followed from one 5-year period to the next to calculate attrition rates as general practitioners and medical specialists left the workforce. The cohorts were formed by grouping the number of general medical practitioners or specialists in the relevant five year age groups at a base year - e.g. all the specialists aged between 25 and 29 years in 1995 were grouped together to form one cohort: the 25-29 year-old specialist cohort. As each cohort consisted of individuals in a five year age group (i.e. 45-49, 50-54, 55-59 years and so on) it was appropriate to calculate attrition of the cohort at 5-yearly intervals so the number of individuals within the cohort who progress from one age group to the next could be determined. Although leaving the workforce may be due to any of a number of factors including retirement, ill health, change of profession, and death, attrition in this paper was broadly grouped as retirement.

Using Medicare and Medical Labour Force Survey data the net attrition or growth rates (that is, the net numbers entering or exiting a cohort) were calculated for general practitioners and specialists every 5 years. This was expressed as the percentage reduction of growth in total numbers of the cohort over the previous 5 years. Cumulative net attrition or growth was the sum of the attrition over all the previous years. The calculation of cumulative attrition rates was as follows:

where CAR = Cumulative attrition rate,

N = number of people,

ti = time period (where, for example, t1 = the year 2000, and t2 = the year 2005), and

t1 = first year of data in the series.

Because there were only 9 years of data for specialists, meaning that attrition could be calculated for the first 5 years and then the next 3 years, the final 2 years of attrition for the second 5 year period was estimated on a pro rata basis from the attrition of the previous 3 years.

The general and specialist medical practitioner data was then 'aged' from a base year of 2000, so that it represented the general and specialist medical practitioner workforce aged 45 and over in 5, 10, 15, 20 and 25 years time. These attrition rates were then applied to younger general practitioners and specialists to project future attrition from the workforce.

A chi-square test of association was undertaken to establish whether the general medical practitioner and specialist workforce had aged significantly between 1985 and 2005. The analyses were undertaken using SAS V9.1 (SAS Institute Inc., Cary, NC, USA).

## Results

### The medical workforce in Australia

#### General medical practitioner numbers and gender 1985-2005

Between 1985 and 2005 the general medical practitioner workforce in Australia grew from 13 831 to 22 262 general medical practitioners. General medical practitioners were predominantly male in all years included in the study, although their majority decreased steadily from 80% in 1985 to 63% in 2005. Women were more highly represented in the younger age groups, accounting for 26% of general medical practitioners aged less than 45 in 1985 compared to 12% of general practitioners aged over 45. By 2005, the proportion of women had grown to 49 and 26% in the younger and older age groups respectively.

#### Specialists numbers and gender 1985-2005

There were about 20 200 specialists in Australia in 1995 increasing to 26 500 by 2003. The majority of specialists were men in 1995 and this was still the case in 2005. However, the proportion of female specialists had increased from 18% to 25%. In the older cohorts, aged 45 years or more, women represented 11% of the specialist workforce in 1995, but increased to 15% in 2003. However, they represented a considerably larger proportion of the younger cohorts, aged less than 45 years, where women represented 26% of the specialist workforce in 1995 and increased to 34% in 2003.

### Ageing of the medical workforce

#### General medical practitioners

The Australian general practitioner workforce has aged significantly since 1985 (p < 0.01), with the proportion of general practitioners aged 45 years and over increasing from 39% to 64%. Male general practitioners were older than their female counterparts, with 70% of men and 52% of women aged over 45 in 2005 (43% and 23% in 1985).

#### Specialists

There has not been marked ageing of the specialist workforce in Australia between 1995 and 2003. However, with only nine years of data, gradual ageing over a longer time period may not be identified. About half of specialists were aged 45 and over in both 1995 and 2003.

### Projected retirement of older general practitioners and specialists

#### General medical practitioners

The attrition rates calculated for general practitioners aged 45 and over were used to project future general medical practitioner retirements from 2005 to 2025. These were based upon the attrition rates of general practitioners and specialists observed between 1985-2005 and 1995-2005 respectively (as shown in Table 1 and Table 2)

**Table 1:** Cumulative attrition from the general physician workforce 1985-2005, Australia

	45-49	50-54	55-59	60-64	65-69	70-74	75-79
**1985**							

**1990**	0%	1%	2%	10%	23%	39%	20%

**1995**	0%	6%	15%	32%	49%	63%	

**2000**	2%	21%	40%	59%	72%		

**2005**	20	48%	67%	78%			

Age groups can be followed down the columns of the table. For example, 10% of the GPs aged 60-64 years in 1985 had left the workforce by 1990, when they were aged 65-69.

Source: Australian Government Department of Health and Ageing Medicare data, 1985 to 2005. Calculation of cumulative attrition rates for each cohort: CAR = 1-(Nti/Nt1) where CAR = Cumulative attrition rate, N = number of people, ti = year in series and t1 = first data year in series (1985).

**Table 2:** Cumulative attrition from the specialist workforce 1995-2005, Australia

	45-49	50-54	55-59	60-64	65-69	70-74	75-79
**1995**							

**2000**	2%	2%	11%	28%	41%	39%	60%

2005*	5%	1%	54%	71%	76%	100%	

*Data for 2004 and 2005 were based on projections assuming the same rate of attrition for the previous 3 years 2001-2003

Age groups can be followed down the columns of the table. For example, 2% of specialists aged 45-49 years in 1995 had left the workforce by the year 2000, when they were aged 50-54.

Source: AIHW Medical Labour Force Survey, 1995 to 2003. Calculation of cumulative attrition rates for each cohort: CAR = 1-(Nti/Nt1) where CAR = Cumulative attrition rate, N = number of people, ti = year in series and t1 = first data year in series (1995). Calculation of attrition from 2000 to 2005 = CAR_2000-2003 _*1 2/3.

**Figure 1:**
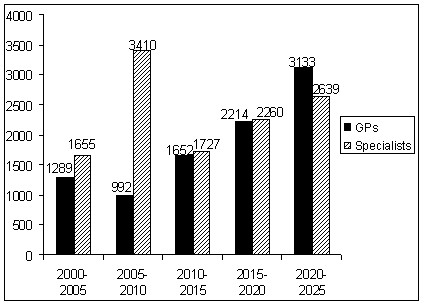
**Projected retirement every five years, general medical practitioners and specialists aged 45 years or more, 2000-2025, Australia**.

**Figure 2:**
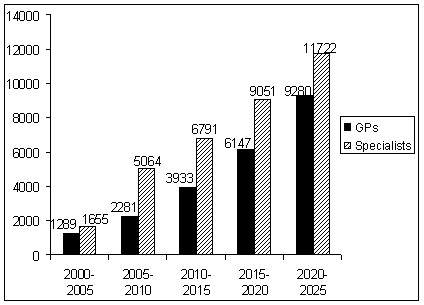
**Projected cumulative retirement every five years, general medical practitioners and specialists aged 45 years or more, 2000-2025, Australia**.

In the year 2000, there were 21 355 general medical practitioners in Australia. Of these, 1289 or 6% were projected to retire by 2005 (Figure 1). A further 5% were expected to retire in the five years to 2010. A greater number of general medical practitioners were expected to leave the workforce in each subsequent five year period so that by 2025, a total of 9280 or 43% of the 2000 workforce would no longer be practicing (Figure 2). The acceleration of retiree numbers is caused by the large group of younger general practitioners in their forties, with the largest group who would reach the age of 65 years in about 20 years time totalling almost 4000 in the 45 to 49 age group.

#### Specialists

Of the specialists who were aged 45 to 49 years in 2005, 39% were projected to cease practicing by 2020 when they were aged 65 to 69 years, with this figure increasing to 64% when they would be 70 to 74 years of age. As with general practitioners, there were some specialists practicing at 80 years of age or more. However as the numbers were too small for reliable projections, it was assumed that all specialists retired after 80 years of age.

Of the approximately 23 300 specialist medical practitioners in the year 2000, about 1655 were projected to retire over the five years to 2005 (Figure 1). A further 3410 were projected to retire in the following five years to 2010, and between 1700 and 2600 every five years after that to 2025. This amounted to a total of 11 722 retirees or 50% of the specialist workforce in the year 2000 (Figure 2). The large number of retirees between 2005 and 2010 reflects the combination of rapid retirement in the 55-69 year age group, which is also shown in the medical labour force historical survey data, (see Table 1) and the large size of the cohort entering these age groups in the second five year period.

### Future medical education workforce

A medical educator, also known as a teacher/educator, is defined by the Australian Institute of Health and Welfare as "a person teaching or training persons in medicine".

The average age of medical educators in 2005 was 50.9 years [11]. This is slightly older than the average age of general medical practitioners and specialists, which in 2005 was 48.6 years and 49.9 years. In twenty years time there will be fewer general medical practitioners in the higher age groups from which medical educators generally belong.

Based upon contemporary numbers of general medical practitioners and specialists, and the growth and subsequent attrition trends of the current medical workforce, some estimates of future workforce numbers in the age groups typical of medical educators can be made. The numbers in the cohort of general medical practitioners who were aged 25-34 in 2005 will increase from the 1161 who are currently in the workforce. In 2025, this cohort will be aged 45-54 and there will be approximately 3340 general practitioners in this age group. This is less than the number of general medical practitioners who were in these age groups in 2005.

The number of specialists in these older age groups could not be projected as far, due to the limited data available. However, it was estimated that in 2013, there will be approximately 8430 specialists in the 45-54 age group, based upon the projected changes to the cohort aged 35-44 years in 2003. This is more than the numbers in this age group in 2003.

## Discussion

The results of this study show that 43% of general medical practitioners and 50% of specialists that were practicing in 2000 will likely have retired by 2025. This represents the loss of a large proportion of the accumulated experience in the general partitioner and specialist health care professional workforce. This upcoming retirement of a large proportion of the experienced general practitioner and specialist workforce may lead to difficulties in meeting the need for an increasing number of future educators, as many of those who have the experience necessary to educate them will have retired.

The ageing of the medical workforce which has been well documented in other studies, is posing numerous workforce planning issues [6,8,12,13]. Training more health care professionals has been seen as one way of overcoming future health workforce shortages potentially caused by this ageing workforce. In 2006, $250 million (Australian dollars throughout) was pledged to be spent on doctor and nurse training to 2010 [10]. There has also been an associated recent increase in the number of training places available, and these new students should begin to fill the 800 to 1300 general practitioner shortages identified by the Australian government in 2005, and at least some of the cohort of retiring practitioners [14]. However, there may be a shortage of experienced general medical practitioners to provide the post graduate training as a large number of experienced workers retires, as demonstrated by this study.

The average age of medical professionals who act as medical educators in 2005 was 50.9 years [11]. This is slightly older than the average age of all general medical practitioners and specialists, which in 2005 was 48.6 years and 49.9 years respectively. It should be noted that some individuals may also practice while also acting as medical educators. In twenty years time (from 2005), there will be fewer general medical practitioners and specialists in the older age groups from which medical educators generally belong. The results of this study indicate that by 2020, 78% of general practitioners aged 45-49 in 2005, and 93% of specialists aged 45-49 in 2005 will have retired from the workforce. This will likely leave a significant shortfall in the experienced health care professionals available to train the larger future health workforce.

It is likely that there will continue to be fewer general medical practitioners acting as medical educators in the future due to the lower pool from which these general medical practitioner educators may be drawn, assuming most of them come from the 45-54 year old age group, as reflected in the mean age of medical educators. The numbers which will be in this age group in 2025 is less than the numbers for this age group in 2005. The number of specialists acting as medical educators is likely to remain unchanged or even increase due to the increasing number of specialists entering this age bracket in the future.

This has been noted by the National Health Workforce Taskforce who identified that there is a "lack of capacity to provide training" for future health care professionals [15]. It also predicted that there would be increasing pressure to place students in training positions in the future as current experienced health care teachers retired.

Furthermore, the lower numbers currently entering the general practitioner workforce will exacerbate the shortage of experienced professionals within this discipline. In 2025, there are expected to be fewer general practitioners in the older age groups than there are at present. Deferring the retirement of these older general practitioners, in order to extend the period of time for which their experience can be utilised in both practice and education, is unlikely to be an option. Their retirement patterns are already characterised by gradual retirement and working beyond the traditional retirement age [8]. The majority of general practitioners and specialists retire over the age of 60, which is much older than the average retirement age for the Australian population of 52 years [16].

Based upon these projections, it seems that there will be a decreased number of experienced general medical practitioners in future years, which will create difficulties in providing education to incoming medical professionals. However, these projections are based upon the assumption that retirement patterns will remain the same as current ones into the future, which can be seen as a limitation of this study. With feminisation of the medical workforce, it is possible that retirement may in the future be somewhat earlier [17].

## Conclusion

Ageing and retirement of the baby boomer and older cohorts of the general medical practitioner and specialist workforce, combined with an ageing population demanding more services, are likely to add to shortages over the next 20 years. Increasing numbers of students and new medical schools has been heralded as a means of alleviating shortages from about 2015 onwards; however, the retirement of a large proportion of experienced health care professionals may lead to shortages of educators of these students.

## Competing interests

The authors declare that they have no competing interests.

## Authors' contributions

DS conceived of the study and participated in the drafting and editing of the manuscript, data analysis and interpretation of the findings. SF participated in the drafting and editing of the manuscript, data analysis and interpretation of the findings. EC participated in the drafting and editing of the manuscript, and interpretation of the findings. All authors read and approved the final manuscript.
